# Choroidal Neovascularization Secondary to Central Serous Chorioretinopathy: OCT Angiography Findings and Risk Factors

**DOI:** 10.1155/2020/7217906

**Published:** 2020-02-07

**Authors:** Joon Hyung Yeo, Richul Oh, Yoon Jeon Kim, June-Gone Kim, Young Hee Yoon, Joo Yong Lee

**Affiliations:** Department of Ophthalmology, Asan Medical Center, University of Ulsan, College of Medicine, Seoul, Republic of Korea

## Abstract

**Purpose:**

To identify the clinical characteristics and risk factors for secondary choroidal neovascularization (CNV) in central serous chorioretinopathy (CSC).

**Methods:**

In this retrospective study, we included a total of 108 eyes in 106 CSC patients. Group A was defined as patients initially diagnosed with CSC who developed secondary CNV, and group B was defined as patients who did not develop secondary CNV. Clinical and demographic characteristics, optical coherence tomography (OCT) findings at CSC diagnosis and OCT angiography (OCTA) at the time of secondary CNV diagnosis, were compared between the groups.

**Results:**

Thirty-one eyes had CNV (group A) and 77 eyes did not (group B). The mean age of group A was higher than that of group B (52.28 ± 6.87 vs. 46.78 ± 9.45 years; *P* < 0.001). Although there was no difference in pigment epithelial detachment (PED) height, group A had larger PED width than group B at CSC diagnosis. The foveal and parafoveal choriocapillary flow densities were significantly lower in group A than group B (*P* < 0.001). Although there was no difference in pigment epithelial detachment (PED) height, group A had larger PED width than group B at CSC diagnosis. The foveal and parafoveal choriocapillary flow densities were significantly lower in group A than group B (*P* < 0.001). Although there was no difference in pigment epithelial detachment (PED) height, group A had larger PED width than group B at CSC diagnosis. The foveal and parafoveal choriocapillary flow densities were significantly lower in group A than group B (

**Conclusion:**

We identified that older age, wider PED width at diagnosis, and recurrent episodes of CSC were independent risk factors for development of secondary CNV. Therefore, patients with these risk factors should be monitored to allow early detection and prompt treatment of secondary CNV.

## 1. Introduction

Central serous chorioretinopathy (CSC) primarily affects middle-aged men and is characterized by serous detachments of the neurosensory retina and retinal pigment epithelium (RPE) [1]. CSC is generally a self-limiting disease associated with a good visual prognosis although subretinal fluid (SRF) may persist for more than 6 months in approximately 5% of cases, [1] resulting in chronic disease characterized by RPE changes and photoreceptor damage [2, 3]. In this long-standing disease, choroidal neovascularization (CNV) may develop, [4] which is a major cause of poor visual prognosis in CSC patients. The prevalence of CNV secondary to CSC ranges from 2% to 15.6% [5–7].

Although the underlying pathophysiologic mechanisms of CSC and secondary CNV remain incompletely understood, some risk factors, including laser photocoagulation or photodynamic therapy (PDT) are known to be related to development of secondary CNV [8]. A few prior studies assessed potential risk factors for CNV secondary to CSC and identified that older age and diffuse RPE loss are risk factors [7, 9].

The purpose of the present study was to improve our understanding of the pathophysiology of CNV in CSC. We therefore sought to identify demographic, systemic, and ocular risk factors for development of CNV in CSC patients. Further, we assessed microvascular changes in CSC patients with and without secondary CNV.

## 2. Methods

All procedures were conducted in accordance with the tenets of the Declaration of Helsinki, and the study design was approved by the Institutional Review Board of Asan Medical Center (IRB no. 2019-0046). Because of the study's retrospective design and use of deidentified patient data, the review board waived the need for written informed consent.

### 2.1. Patients

A retrospective consecutive chart review was conducted for all patients initially diagnosed with CSC at the retina clinic of Asan Medical Center and for those who underwent OCTA between December 2016 and November 2018 during follow-up. CSC was diagnosed based on a history of blurred or distorted central vision, central scotoma, and/or micropsia. Comprehensive ophthalmologic examinations, including a review of the patients' medical and clinical histories, measurement of best-corrected visual acuity (BCVA), slit-lamp biomicroscopy, dilated fundoscopic examination, optical coherence tomography (OCT), and dye-based angiography, were conducted. When dye-based angiography revealed the presence of CNV upon initial examination, the case was diagnosed as either chronic CSC with CNV, age-related macular degeneration (AMD), or idiopathic CNV and was not included in this study. In addition, subjects were excluded if any of the following were present: (1) previous history of CSC that had not been verified in our clinic; (2) failure to achieve complete resolution of SRF; (3) absence of OCTA images after resolution of SRF; (4) concomitant ocular diseases other than cataracts, including pre-existing glaucoma, AMD, epiretinal membrane, retinal vein occlusion, diabetic retinopathy, steroid-induced CSC, or CNV secondary to other retinal diseases; (5) high myopia (refractive error (spherical equivalent) of −6 diopters or greater); (6) history of vitrectomy.

Data collected from patient records included age, sex, systemic diseases, BCVA, treatment modalities, number of treatments, baseline OCT findings, fluorescein angiography (FA) and indocyanine green angiography (ICGA) findings, and OCTA findings. BCVA in Snellen value was converted to the logarithm of the minimum angle of resolution for statistical analyses.

### 2.2. Optical Coherence Tomography

At the time of initial examination (baseline), all patients underwent spectral domain OCT (Spectralis; Heidelberg Engineering Inc., Carlsbad, California) using the AutoRescan mode. The AutoRescan feature of the Spectralis OCT device provides an OCT scan and a corresponding high-quality fundus picture, and it relies on active eye tracking. OCT images were generated using a horizontal spectral domain OCT cross section (25 lines spaced 240 *μ*m apart). To improve image quality, 25 to 30 frames were averaged for each B-scan.

The OCT parameters (maximum width and height of pigment epithelial detachment (PED), maximum width and height of subretinal fluid (SRF), subfoveal choroidal thickness (SCT), and the presence of intraretinal fluid (IRF)) were measured manually by two independent investigators (J.H.Y. and R.O.) using Heidelberg Eye Explorer Software version 1.7.0.0 (Heidelberg Engineering Inc.). One B-scan image with the maximum width or height of PED/SRF was chosen for measurement. The SCT was measured on central horizontal raster EDI scans, from the outer portion of the RPE line to the inner surface of the sclera. Each investigator took five measurements from one image and discarded the maximal and minimal values. The mean value of six measurements was used in subsequent analyses.

Central subfield thickness (CST) was defined as the average thickness of the macular in the central 1 mm ETDRS grid. The CST was automatically measured with the instrument's internal software.

Chronic CSC was defined by the presence of SRF documented on OCT imaging for at least 6 months within the first episode. Persistent PED was not considered as chronic CSC. Recurrence of CSC was defined as documented resolution of SRF followed by reappearance of SRF in the same eye.

### 2.3. Optical Coherence Tomography Angiography

An Optovue RTvue XR Avanti with Angiovue was used to acquire the OCTA image. A scan area of 3 × 3 mm^2^ centered on the fovea was chosen. Each resulting OCTA en face image contained 304°×°304 pixels created from the intersection of the 304 vertical and 304 horizontal B-scans. Each full-thickness retinal scan was segmented as follows: superficial capillary plexus (SCP) from the inner limiting membrane to the outer limits of the inner plexiform layer (IPL), deep capillary plexus (DCP) from the outer limits of the IPL to the outer limits of the outer plexiform layer (OPL), and choriocapillaris from the RPE line to 31 *μ*m below the line. Flow density was calculated as the percentage area occupied by vessels in the selected region. The foveal flow density (FFD) and parafoveal flow density (PFD) were defined as the flow density in the foveal region with a diameter of 1 mm and that in the parafoveal region with a diameter of 1–3 mm, respectively.

All scans were reviewed independently by two investigators (J.H.Y. and R.O.) who were blinded to all clinical information to ensure correct segmentation and sufficient quality. In the case of incorrect segmentation, we manually adjusted the boundary using the Angiovue module of Optovue RTvue XR Avanti software installed on the instrument. We defined images of sufficient quality as those centered on the macula and without significant motion artifacts or edge duplication. Cases with insufficient image quality were excluded from the study.

**Figure 1:**
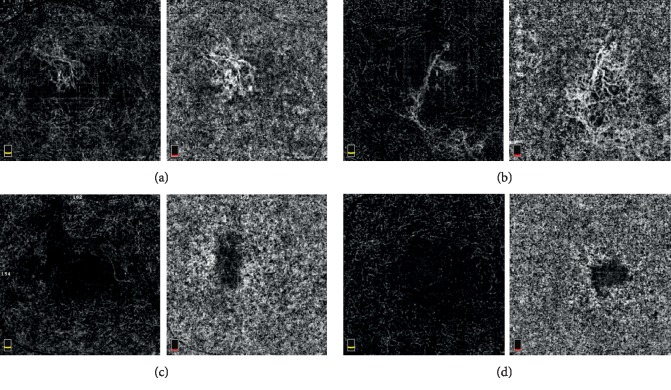
Representative optical coherence tomography angiography (OCTA) images of (a and b) patients with secondary choroidal neovascularization (CNV) and (c and d) patients without CNV. In patients with CNV secondary to CSC, an aberrant flow signal was detected by OCTA in the outer retina and choriocapillaris.

OCTA images of all included eyes in this study were acquired after complete resolution of SRF during follow-up. CNV was defined as flow signal in the outer retina, aberrant flow signal in the choriocapillaris consistent with known morphologic features of CNV, or both. Based on the presence of CNV, patients were divided into two groups: patients in whom CNV was confirmed by OCTA (group A), and patients who did not have CNV secondary to CSC (group B) (Figure 1). The presence of CNV was determined by consensus between two investigators (J.H.Y. and R.O.) in a blinded fashion. If there was disagreement between the investigators, a third blinded investigator (Y.J.K.) will adjudicate the decision.

### 2.4. Statistical Analysis

All statistical analyses were performed using SPSS version 20.0 (SPSS Inc., Chicago, IL, USA). Continuous variables are presented as mean ± standard deviation. Intergroup comparisons were evaluated using a Student's *t*-test, a Wilcoxon rank-sum test, or a chi-squared test as appropriate. Multivariate logistic regression analysis was used to determine factors significantly associated with development of CNV. *P* values less than 0.05 were considered statistically significant.

## 3. Results

The study included a total of 108 eyes from 106 CSC patients (81 eyes in 79 men and 27 eyes in 27 women). At initial examination, ICGA revealed dilated choroidal vessels and choroidal hyperpermeability, not CNV, in all patients. Among them, 31 eyes in 29 patients were diagnosed with CNV (group A) after complete resolution of SRF, while the other 77 eyes in 77 patients did not have CNV (group B). The mean age of group A was 52.28 ± 6.87 years (range, 42–69 years) and that of group B was 46.78 ± 9.45 years (range, 30–68 years). This difference was statistically significant (*P* < 0.001). Group A had higher incidence of systemic hypertension (24.14%) than group B (11.69%), but this difference was not statistically significant (*P*=0.196). Patient characteristics are summarized in Table 1.

### 3.1. Ocular Characteristics

BCVA (logMAR) at initial examination and at the time of CNV diagnosis were better in group B than in group A, but this difference was not statistically significant (*P*=0.192 at initial exam; *P*=0.158 at final exam). Although both groups demonstrated a slight improvement through the course of follow-up, there was no significant difference in BCVA change between the groups (*P*=0.435). Recurrent episodes of CSC were more commonly observed in group A, which was statistically significant (*P*=0.032). Chronic CSC occurred in 83.87% of patients in group B and 70.13% of patients in group A (*P*=0.156; Table 1).

### 3.2. Baseline OCT

**Figure 2:**
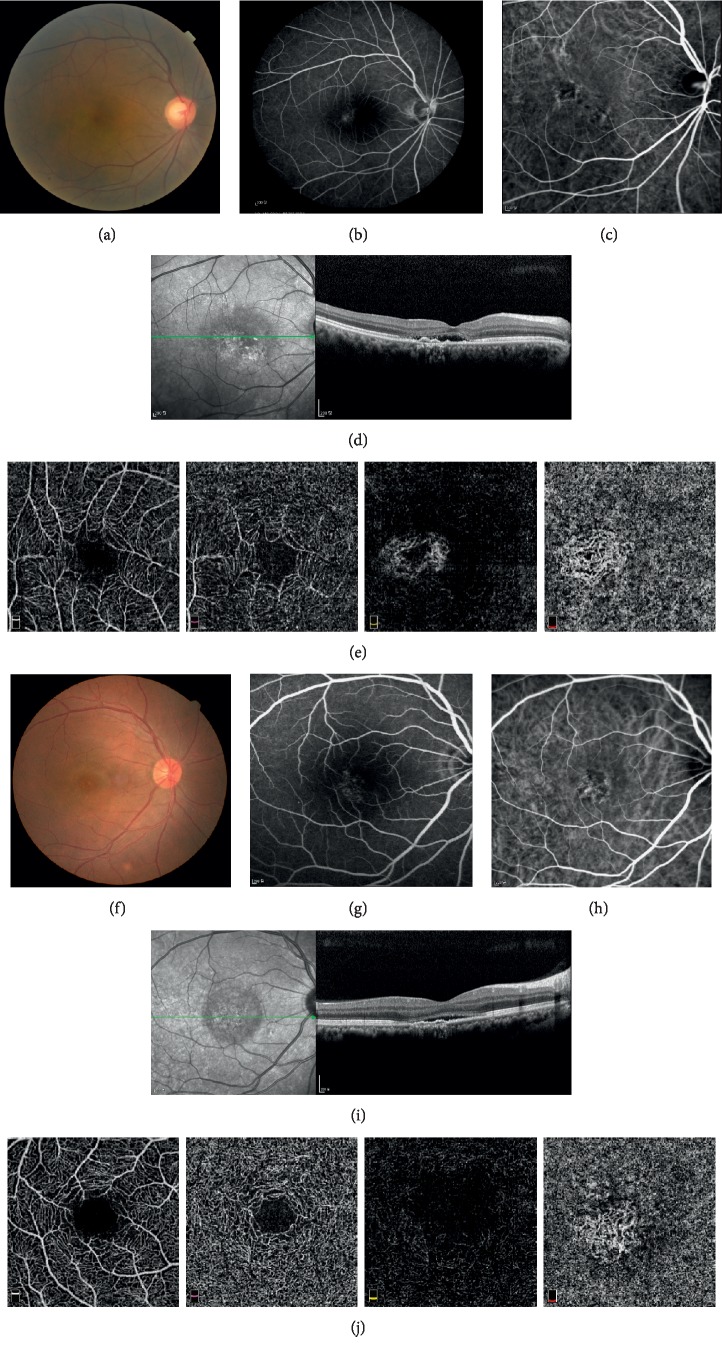
Representative fundus images, dye-based angiography, and optical coherence tomography (OCT) images at the time of initial examination (baseline), and OCTA images of (a–e) the eye of a 54-year-old man and (f–j) the eye of a 43-year-old man. Focal leakage on fluorescein angiography (b and g), choroidal vessel dilation on indocyanine green angiography (c and h), and subretinal fluid with pigment epithelial detachment (d and i) (width: 971 *μ*m and 1457 *μ*m, respectively) on OCT. During the follow-up (80 and 20 months), OCTA revealed the presence of CNV (e and j).

Table 2 summarizes data obtained from baseline OCT. All patients had variable SRF size, but there was no statistically significant difference in either SRF width or height between the groups. Although PED presence showed no statistically significant difference, the size of PED significantly differed between the groups. Although there was no difference in PED height between the two groups, group A had a much greater PED width than group B (Figure 2). Therefore, we calculated a best cutoff value for PED width in relation to development of CNV. With a PED width cutoff value of 939 *μ*m, we could effectively divide the cohort of patients into CNV and non-CNV groups (AUC: 0.845; sensitivity: 0.690; specificity: 0.846). However, SCT and CST showed no difference between the groups. Also, intraretinal fluid occurred in only one eye, which did not develop CNV.

### 3.3. Factors Related to CNV Development

We used logistic regression analysis with a generalized estimation equation model to identify risk factors related to development of CNV. The results of univariate and multivariate analyses are shown in Table 3. Multivariate logistic regression analysis identified that older age (OR, 1.080; 95% confidence interval (CI), 1.021–1.143; *P*=0.007), wider PED (OR, 12.101; 95% CI, 2.035–71.945; *P*=0.006), and one or more recurrent episodes of CSC (OR, 3.084; 95% CI, 1.077–8.831; *P*=0.036) were risk factors for CNV. Other factors were not found to be risk factors.

### 3.4. Differences in Choriocapillaris Flow Density between Groups

During follow-up, OCTA was acquired in group A after an average of 51.48 months and in group B after an average of 29.14 months, following initial diagnosis.  Table 4 shows the flow density data of both groups, as detected by OCTA. Eyes with CNV had decreased total choriocapillaris flow density relative to eyes without CNV (*P* < 0.001), as well as decreased foveal choriocapillaris flow density (*P*=0.027) and decreased parafoveal choriocapillaris flow density (*P* < 0.001). Choriocapillaris parafoveal flow in particular was much lower in eyes with CNV. When we subdivided the parafovea into four quadrants, choriocapillaris flow density was decreased in CNV eyes in all four quadrants (*P*=0.002, *P* < 0.001, *P*=0.001, and *P* < 0.001, respectively).

## 4. Discussion

In this study, we identified older age, wide PED width at diagnosis, and recurrent episodes of CSC as independent risk factors for the development of CNV secondary to CSC. In addition, using OCTA, we found that CSC patients with secondary CNV had lower choriocapillary flow densities than those without secondary CNV.

Dye-based angiography has been the gold standard for CNV diagnosis for several decades, [10] but dye-based imaging is invasive and can result in nausea, vomiting, and anaphylactic reactions. Moreover, the definitive diagnosis of CNV in CSC using dye-based angiography may be challenging due to overlap in clinical presentations and image findings. The advent of OCTA has enabled visualization of choriocapillaris and other vascular signal abnormalities at the choriocapillary level in CSC patients [11]. As in the previous study, we analyzed OCTA findings to detect CNV and divided patients into two groups (with or without CNV). Subsequently, CNV was detected in 31 of 108 eyes (28.70%), which is higher than the prevalence reported previously. The ability of OCTA to detect early microvascular changes might have been responsible for the higher prevalence of secondary CNV in this study. A recent OCTA study suggested that OCTA has improved sensitivity and specificity over FA for the detection of CNV in eyes with CSC [12]. In addition, Palejwala et al. [13] reported the applicability of OCTA for early detection of CNV. In their series, they found that OCTA could detect early CNV (type I), which was difficult to be detected using conventional FA and OCT. Therefore, we believe that the prevalence of CNV in this study may be different or even higher than that of previous studies that used FA and/or ICGA.

Although secondary CNV in CSC is reported in several studies, [4, 5, 7, 14] only a few studies investigated risk factors for development of CNV in patients with CSC. Previous studies suggest that CNV may be more prevalent in patients over 50 years of age with chronic CSC [7, 15]. In the present study, CNV occurrence was 40.38% (21/52) in patients over 50 years of age and 17.86% (10/56) in patients under 50 years of age, which was statistically significant. Moreover, multivariate logistic regression analysis revealed that older age was a risk factor for CNV secondary to CSC, which is in agreement with prior studies that suggest that the prevalence of CNV in CSC is much higher among elderly patients [7].

The present study further demonstrated that CSC accompanied by PED at the initial diagnosis was associated with an approximately 12-fold increased risk of secondary CNV. More specifically, a PED width >939 *μ*m at baseline was a risk factor for development of secondary CNV. PED is well known to be associated with AMD, CSC, and polypoidal choroidal vasculopathy [14, 16]. In addition, recent studies identified that double-layer signs, which corresponded to flat irregular PED, are associated with development of CNV secondary to CSC [17, 18]. In the present study, variable shapes and sizes of PEDs were observed and differentiated through OCT imaging. Using OCTA, we detected CNV in 61.70% (29/47) of eyes with flat irregular PED, which was in partial agreement with prior findings. Although flat irregular PED was detected in approximately 50% of eyes with CSC, no definitive sign of CNV was detected by dye-based angiography at baseline in the patients included in the study. Accordingly, neovascular activity of flat irregular PED at the initial phase could be considered low grade. However, flat irregular PED may manifest as the active form of CNV at the chronic phase. Hage et al. evaluated clinical findings in patients with chronic CSC to distinguish flat irregular PED from type 1 CNV [14]. The authors reported that opacity of the subepithelial content is a potential indicator of CNV and suggested that combining information obtained using different imaging modalities could be the optimal approach for diagnosis of secondary CNV. We therefore suggest that flat irregular PED (especially with a width over 939 *μ*m) with opaque content should be noted by clinicians as a potential indicator of CNV and multimodal imaging should be used in these cases.

Many prior studies revealed that chronic CSC is predisposed to development of secondary CNV [4, 5, 19]. However, in the present study, we demonstrated that recurrent episodes of CSC, but not chronic CSC, were a risk factor for development of secondary CNV. Because of varying definitions, methods, and lengths of follow-up, rates of recurrence in previous studies are difﬁcult to directly compare with the present study. Nonetheless, the recurrence rate in the present study (44.44%) is within the range found in other studies (15.4–50.7%) [2, 5, 20, 21]. Although the exact pathophysiology of CSC is still poorly understood, advances in imaging techniques, particularly ICGA, OCT, and OCTA, have led to a better understanding of the pathophysiology of this disease. It was reported that choroidal ischemia and increased hydrostatic pressure in the choroidal network create secondary damage in the RPE that leads to the breakdown of the external blood-retinal barrier, which might contribute to the development of secondary CNV [22]. Lee et al. identified that RPE atrophy with pigmentary changes, a well-known indicator of long-standing CSC, is a risk factor for secondary CNV in eyes with prior CSC episodes [17]. Although we did not evaluate RPE changes in the present study, our finding that recurrent episodes of CSC were associated with increased risk of CNV is in accordance with prior findings suggesting that long-standing CSC is associated with secondary CNV. Therefore, we believe that recurrent episodes of choroidal ischemia can lead to the development of secondary CNV although this is the only speculation and there are probably other potential explanations. However, the follow-up duration of group B with no CNV was relatively short. Therefore, our findings in this respect should be interpreted cautiously.

Secondary CNV is a well-known complication of PDT and laser photocoagulation used to treat CSC [8]. In the present study, 16 of 31 eyes (51.61%) in group A and 37 of 77 eyes (48.05%) in group B underwent PDT or focal laser treatment, which showed no significant difference. Because PDT can potentially reduce choroidal perfusion and thus increase the risk of secondary CNV, PDT protocols using half fluence in our clinic may contribute to successful treatment outcomes for CSC without increasing the risk of secondary CNV.

Although OCTA detection of CNV secondary to CSC is well documented, [12, 23, 24] no prior studies used OCTA to quantify choriocapillaris flow density in CSC patients. The present study identified reduced choriocapillaris flow density in CSC eyes with secondary CNV, while retinal circulation did not differ between the groups. To minimize the masking artifact that might have skewed the results, we acquired OCTA images after complete resolution of SRF during follow-up. Although CNV might cause a masking artefact, as shown in Table 4, group A had decreased parafoveal flow density in all four quadrants as well as decreased foveal flow density, regardless of location of the CNV. The association of decreased choriocapillaris flow density with CNV is consistent with previous studies that used OCTA to investigate CNV in exudative AMD [25, 26]. Mechanical stress caused by focal or diffuse enlarged underlying choroidal vessels is thought to reduce choriocapillaris flow by compressing the choriocapillaris, [27, 28] resulting in choroidal ischemia that could contribute to development of secondary CNV [29–31]. However, the present study did not reveal an SCT as a risk factor for secondary CNV.

The present study has several limitations. First, no unified criteria were used for CNV detection. After SRF resolution, we defined CNV based on the findings of OCTA images, while dye-based angiography was performed to document the absence of CNV at initial diagnosis. Although it was suggested that OCTA may be a viable alternative to dye-based angiography in the diagnosis of CNV, identification of CNV only by OCTA without FA and/or ICGA might have skewed the results. It is also possible that CNV patients with low neovascular activity not visible on dye-based angiography at the time of CSC diagnosis were included in the study. Second, this study had a retrospective design and thus selection bias could have accentuated some risk factors and masked others. In addition, we were unable to assess the precise cause and effect relationship between microvascular changes and development of secondary CNV. Further prospective studies investigating whether microvascular changes are associated with development of secondary CNV will further elucidate the pathophysiology of CNV secondary to CSC. Third, OCTA artifacts could have compromised our results. Fourth, the sample size was relatively small. However, to the best of the author's knowledge, the cohort included the largest number CSC/CNV patients yet to be analyzed in the literature. Finally, all patients were Asian (Korean), so our data may not be automatically generalizable to other ethnicities.

In conclusion, we found that older age, wider PED width at diagnosis, and recurrent episodes of CSC were significantly associated with development of secondary CNV in CSC. We also identified lower choriocapillaris flow density in patients with secondary CNV compared with patients without CNV. Although secondary CNV is a relatively uncommon complication of CSC, this complication severely limits visual prognosis. Therefore, eyes with wide PED, as revealed by baseline OCT, or eyes with recurrent CSC episodes should be carefully examined for early detection of CNV. Early detection followed by prompt treatment will improve visual outcomes in CNV secondary to CSC.

## Figures and Tables

**Table 1:** Demographics and clinical characteristics of study patients.

	Total patients (*n* = 106)	Group A (*n* = 29)	Group B (*n* = 77)	*P*
Eyes, *n* (%)	108 (100)	31 (28.70)	77 (71.30)	
Mean age, years	48.45 (9.13)	52.28 (6.87)	46.78 (9.45)	<0.001^*∗*^
Male, *n* (%)	79 (74.53)	20 (68.97)	59 (76.62)	0.578^†^
HTN, *n* (%)	16 (15.09)	7 (24.14)	9 (11.69)	0.196^†^
DM, *n* (%)	6 (5.66)	0 (0.00)	6 (7.79)	0.282^†^
BCVA, logMAR				
Initial	0.275 (0.279)	0.342 (0.365)	0.248 (0.233)	0.192^‡^
Final	0.167 (0.176)	0.206 (0.188)	0.151 (0.170)	0.158^‡^
Follow-up duration, months	35.56 (37.75)	51.48 (47.42)	29.14 (31.21)	0.006^‡^
Recurrence, *n* (%)				0.032^†^
Recurrence	48 (44.44)	19 (61.29)	29 (37.66)	
No recurrence	60 (55.56)	12 (38.71)	48 (62.34)	
Chronicity, *n* (%)				0.156^†^
Yes	80 (74.07)	26 (83.87)	54 (70.13)	
No	28 (25.93)	5 (16.13)	23 (29.87)	
Treatment, *n* (%)				
Anti-VEGF	73 (67.59)	22 (70.97)	51 (66.23)	0.592^†^
PDT or focal laser	55 (50.93)	16 (51.61)	37 (48.05)	0.903^†^

Data are presented as mean (standard deviation) unless otherwise indicated. ^*∗*^Student's *t*-test; ^†^chi-squared test; ^‡^Wilcoxon rank-sum test. HTN, hypertension; DM, diabetes mellitus; BCVA, best-corrected visual acuity; logMAR, logarithm of minimal angle of resolution; anti-VEGF, antivascular endothelial growth factor; PDT, photodynamic therapy.

**Table 2:** Optical coherence tomography parameters of study eyes.

	Total (*n* = 108)	Group A (*n* = 31)	Group B (*n* = 77)	*P*
CST, *µ*m	406.24 (129.46)	406.84 (114.18)	406.00 (135.82)	0.709^*∗*^
PED, *n* (%)				0.336^†^
Yes	94 (87.04)	29 (93.55)	65 (84.41)	
No	14 (12.96)	2 (6.45)	12 (15.58)	
PED height, *µ*m	58.99 (57.69)	57.52 (28.61)	59.58 (66.04)	0.216^*∗*^
PED width, *µ*m	671.53 (507.12)	1107.13 (538.97)	496.16 (372.62)	<0.001^*∗*^
SCT, *µ*m	389.64 (101.71)	376.90 (84.79)	394.76 (107.86)	0.622^*∗*^
SRF height, *µ*m	206.78 (122.87)	187.42 (98.41)	214.57 (131.21)	0.429^*∗*^
SRF width, *µ*m	2712.70 (1344.70)	2622.94 (908.59)	2748.84 (1488.39)	0.644^*∗*^
IRF, *n* (%)				1.00^†^
Yes	1 (0.93)	0 (0)	1 (1.30)	
No	107 (99.07)	31 (100)	76 (98.70)	

Data are presented as mean (standard deviation) unless otherwise indicated. ^*∗*^Student's *t*-test; ^†^chi-squared test. CST, central subfield thickness; PED, pigment epithelial detachment; SCT, subfoveal choroidal thickness; SRF, subretinal fluid; IRF, intraretinal fluid.

**Table 3:** Odds ratios from univariate and multivariate logistic regression analyses of factors associated with occurrence of CNV secondary to CSC.

Variables	Univariate		Multivariate	
	Odds ratio (95% confidence interval)	*P*	Odds ratio (95% confidence interval)	*P*
Patient variables				
Age	1.079 (1.030, 1.131)	0.001^*∗*^	1.080 (1.021, 1.143)	0.007^*∗*^
Sex, female	1.341 (0.516, 3.484)	0.547		
HTN (yes)	2.204 (0.731, 6.647)	0.161		
DM (yes)	N/A	N/A		
Lesion variables				
Initial BCVA (logMAR)	3.134 (0.875, 11.223)	0.079		
Final BCVA (logMAR)	5.798 (0.626, 53.715)	0.122		
SCT	0.998 (0.994, 1.002)	0.370		
≥346.5 *µ*m	1.615 (0.660, 3.955)	0.294		
CST	1.000 (0.997, 1.003)	0.974		
SRF width	1.000 (1.000, 1.000)	0.585		
SRF height	0.998 (0.995, 1.001)	0.254		
PED width	1.003 (1.002, 1.004)	<0.001^*∗*^		
0	1	<0.001^*∗*^	1	<0.001^*∗*^
<939 *µ*m	0.982 (0.188, 5.136)	0.983	1.299 (0.225, 7.510)	0.770
≥939 *µ*m	12.000 (2.240, 62.283)	0.004^*∗*^	12.101 (2.035, 71.945)	0.006^*∗*^
PED height	0.999 (0.994, 1.005)	0.813		
0	1	0.095		
<41.5	1.364 (0.229, 8.121)	0.733		
≥41.5	3.349 (0.685, 16.364)	0.135		
IRF (yes)	N/A	N/A		
Chronicity (yes)	2.215 (0.748, 6.554)	0.151		
Recurrence (yes)	2.771 (1.125, 6.825)	0.027^*∗*^	3.084 (1.077, 8.831)	0.036^*∗*^

^*∗*^Indicates significant *P* value (*P* < 0.05).

**Table 4:** Optical coherence tomography angiography parameters of study eyes.

	Total (*n* = 108)	Group A (*n* = 31)	Group B (*n* = 77)	*P*
Whole image FDs, %	62.11 (4.58)	59.44 (4.21)	63.19 (4.30)	<0.001^*∗*^
FFD, %	63.42 (6.22)	61.64 (5.55)	64.14 (6.37)	0.027^*∗*^
PFD, %	61.16 (4.99)	58.30 (4.55)	62.32 (4.72)	<0.001^†^
Superior, %	61.94 (4.71)	59.66 (4.56)	62.86 (4.48)	0.002^*∗*^
Temporal, %	60.62 (6.24)	57.16 (5.51)	62.02 (6.00)	<0.001^*∗*^
Nasal, %	60.15 (6.03)	57.03 (6.17)	61.41 (5.53)	0.001^*∗*^
Inferior, %	61.92 (5.18)	59.29 (4.88)	62.98 (4.94)	<0.001^*∗*^

Data are presented as mean values (standard deviation). ^*∗*^Student's *t*-test; ^†^Wilcoxon rank-sum test. FD, flow density; FFD, foveal flow density; PFD, parafoveal flow density.

## Data Availability

The data used to support the findings of this study are available from the corresponding author upon request.

## Additional Points

Summary statement: older age, wider pigment epithelial detachment width at central serous chorioretinopathy (CSC) diagnosis, and recurrent episodes were identified as independent risk factors for development of secondary choroidal neovascularization in CSC.
